# Ethical Considerations of Using Machine Learning for Decision Support in Occupational Health: An Example Involving Periodic Workers’ Health Assessments

**DOI:** 10.1007/s10926-020-09895-x

**Published:** 2020-06-04

**Authors:** Marianne W. M. C. Six Dijkstra, Egbert Siebrand, Steven Dorrestijn, Etto L. Salomons, Michiel F. Reneman, Frits G. J. Oosterveld, Remko Soer, Douglas P. Gross, Hendrik J. Bieleman

**Affiliations:** 1 School of Health, Saxion University of Applied Sciences/AGZ, M.H. Tromplaan 28, 7500 KB Enschede, The Netherlands; 2 Research Group Ethics & Technology, Saxion University of Applied Sciences, Enschede, The Netherlands; 3 School of Ambient Intelligence, Saxion University of Applied Sciences, Enschede, The Netherlands; 4 Department of Rehabilitation Medicine, University Medical Center Groningen, University of Groningen, Groningen, The Netherlands; 5 University Medical Center Groningen, Pain Centre, University of Groningen, Groningen, The Netherlands; 6 Department of Physical Therapy, University of Alberta, Edmonton, Canada; 7 University of Groningen, Groningen, The Netherlands

**Keywords:** Machine learning, Clinical decision support system, Occupational health, Ethics, Morals, Evidence based practice

## Abstract

*Purpose* Computer algorithms and Machine Learning (ML) will be integrated into clinical decision support within occupational health care. This will change the interaction between health care professionals and their clients, with unknown consequences. The aim of this study was to explore ethical considerations and potential consequences of using ML based decision support tools (DSTs) in the context of occupational health. *Methods* We conducted an ethical deliberation. This was supported by a narrative literature review of publications about ML and DSTs in occupational health and by an assessment of the potential impact of ML-DSTs according to frameworks from medical ethics and philosophy of technology. We introduce a hypothetical clinical scenario from a workers’ health assessment to reflect on biomedical ethical principles: respect for autonomy, beneficence, non-maleficence and justice. *Results* Respect for autonomy is affected by uncertainty about what future consequences the worker is consenting to as a result of the fluctuating nature of ML-DSTs and validity evidence used to inform the worker. A beneficent advisory process is influenced because the three elements of evidence based practice are affected through use of a ML-DST. The principle of non-maleficence is challenged by the balance between group-level benefits and individual harm, the vulnerability of the worker in the occupational context, and the possibility of function creep. Justice might be empowered when the ML-DST is valid, but profiling and discrimination are potential risks. *Conclusions* Implications of ethical considerations have been described for the socially responsible design of ML-DSTs. Three recommendations were provided to minimize undesirable adverse effects of the development and implementation of ML-DSTs.

## Introduction

Machine Learning (ML) techniques have become part of our daily lives, with examples such as tailored online shopping on Amazon or the social fitness network STRAVA (www.strava.com) which is fully supported by algorithms to track and share cycling and running exercises. The introduction of ML for occupational health care seems inevitable [1, 2]. An insight from the philosophy of technology is that new technologies change our behaviours and our understanding of the world around us [3]. Implementation of an ML-based Decision Support Tool (ML-DST) could drastically change the way occupational health care providers (OHCP) work, make decisions, and interact with clients. It also has implications for how OHCP and their clients perceive and value the changes.

The clinical scenario in Table 1 about ML for decision support in preventive occupational health care raises questions such as, “how will ML influence health-related choices of the worker?” and “will the ML technique result in discrimination?”. In philosophical and ethical approaches to technology assessment, it is proposed that scenarios are a good way to explore the soft impact (i.e. impact that cannot be quantified) of technology before it is fully functional [4, 5]. It helps to anticipate possible adverse effects with ethical implications and therefore allows for more socially responsible innovation [6, 7]. As ML-DSTs are being developed for use in health care settings, it seems appropriate to refer to the four pre-dominating biomedical ethical principles presented by Beauchamp and Childress [8]: respect for autonomy, justice, beneficence and non-maleficence.

**Table 1:** Clinical scenario

Tom (54 years old, team-leader at a car plant) wants to improve his health because he feels tired and has gained too much weight lately. His employer wants to support him so he can be more productive and efficient in both future work and private life. An Occupational Health Care Professional (OHCP) is hired by the company to advise employees on strategies for achieving sustainable employment. Tom consults the OHCP. After Tom completes an occupational health assessment, the OHCP prints a list of potential interventions from a computer and reads it to Tom. The advice was generated by a Decision Support Tool that is based on data of many employees from a variety of companies. The Decision Support Tool uses Machine Learning algorithms for decision making. Tom feels aversion that the computer tells him what to do and resistant to the recommended mental health intervention; “I am not mentally ill, I just feel a bit tired of the recent reorganization”. Now Tom becomes afraid of what the outcome will mean for his function and employment in the company. Due to a precarious financial position the company is very selective about who they employ and Tom thinks mental health issues could implicate job vulnerability. Besides, how will the insurance company act when they find out? On the other side of the table, the OHCP is also surprised by the proposed mental health interventions; based on his conversation with Tom and his professional intuition, he would have proposed something different. He does agree with the advised physical training program and dietary intervention. The OHCP wonders if he should convince Tom, because motivating clients towards a healthier lifestyle is one of the OHCP’s competences. Before that, he would need to convince himself that the advice is correct. When Tom returns to the work-floor he talks to his colleague Jack about the algorithm-based diagnosis and recommendations. Jack is upset because he assumes that his own assessment results could have been used for the algorithm and this is not what he wanted when he agreed to use his data for scientific purposes. However, the assessment had a positive effect on his own health and he has remained employed largely because of it	

The aim of this study is to explore potential consequences and ethical considerations of using ML-DSTs in the context of occupational health. These insights raise awareness for users (OHCPs) as well as technology developers and guiding points to consider during responsible design of ML-DSTs. The ethical deliberation is elaborated using our example from the context of health prevention, namely a workers’ health assessment (WHA) done periodically to predict the workers’ likelihood of developing a health disorder. However, the ethical issues discussed apply equally to other occupational health-related procedures, such as return to work assessments or rehabilitation interventions [2, 9].

## Methods

### Study Design

We conducted an ethical deliberation. This was supported by a narrative literature review of publications about ML-DSTs and by an assessment of the impact of ML-DSTs according to frameworks from medical ethics [8] and the philosophy of technology [5, 10–12].

### Literature Review

A narrative literature review was conducted focusing on ethical considerations of using ML techniques for decision support in health care settings. Relevant terms and definitions used to indicate the field were searched for using library health databases (PubMed and Medline) and Google Scholar [13]. Search terms included: big data analyses, machine learning techniques, artificial intelligence and machine intelligence in combination with decision support, health and ethics. A snowball method was used after the first search.

### Ethical Principles and Impact Assessment

Most ML-related literature is not specifically focused on occupational health. Therefore, knowledge from the fields of technology assessment and moral philosophy of technology [5, 10–12] was applied to consider the impact and potential adverse effects of ML in the occupational context. We focused on concepts used explicitly and implicitly in the available texts, such as adverse effects (negative side effects), behaviour steering, and views on technology. Moreover, we reflected on the widely accepted four major values in biomedical ethics as formulated by Beauchamp and Childress [8]: respect for autonomy, beneficence, non-maleficence and justice. The values are defined and explained in Table 2. These are the predominant values in western society [14] and might be affected by using DSTs.

**Table 2:** The four predominating biomedical ethical principles by Beauchamp and Childress [8]

*Respect for autonomy* addresses the duty for a professional to respect someone’s decision-making capacity and enabling individuals to make reasoned informed choices. In practice this means that the professional has to reduce limiting factors in decision making. In a more positive way this means that there is an obligation to disclose information, giving options and helping individuals gain understanding. There is also the obligation to respect a decision once it is made	
*Beneficence* is the principle of acting with the best interest of the other in mind. More specifically, within healthcare settings, the health care professional should act in a way that benefits the client. Examples are promoting health, reducing risks and preventing health issues and disease. The principle also considers balancing the benefits of interventions against risks and costs	
*Non*-*maleficence* is the principle of “above all, do no harm”. Harm is not just inflicting physical or mental pain and suffering, but can also be seen as going against one’s needs and interests. Although all treatment involves some potential for harm, the harm should be proportionate to the benefit of the treatment	
*Justice,* which can be divided into: (1) justice as equality regarding benefits, risks and costs; and (2) justice as a fair distribution of rights and duties [15]. Specifically this means that a health professional has the obligation to be unbiased	

### Examples Used for the Deliberation

The clinical scenario is used as an example for the ethical considerations [4, 5, 12]. WHA and ML are now briefly explained, because this provides essential context to the scenario. The ethical consequences of *using* an ML-DST are considered primarily from the worker’s perspective. However, the professional’s perspective and developmental aspects that effect responsible use are also considered.

#### Worker’s Health Assessment (WHA)

A WHA is a screen by an OHCP to identify health risks in order to prevent absenteeism, presenteeism, and promote sustained employability [16–19]. With regard to prevention of health conditions that influence work ability, evidence-based resources have been implemented into periodic WHAs [16, 20]. Within WHAs, biometrics (e.g., blood-pressure, cholesterol, glucose, Body Mass Index, waist circumference), functional capacity measurements, and questionnaires measuring relevant constructs (e.g., workability, engagement, vitality, mental and physical stress and strain, health status, lifestyle) may be administered for health and work-related risk assessments. After the assessment, the OHCP provides personalized advice and recommendations for interventions, if applicable. The employer may be informed on anonymous group level about the results, but individual worker results and recommendations are typically not shared with employers. Often various health-related interventions are recommended.

#### Machine Learning (ML) and Data Handling

ML has potential for designing improved DSTs for sustained employability and is an analytical solution to overcome the limitations of traditional best evidence approaches [21–23]: combining different types of measures, pattern recognition, processing large volumes of data, and learning during the process [13].

In the introduction of his special series [13] the authors describe ML as focusing “on optimizing relevant performance metrics via learning on a training set and testing on a validation set. In this set up, the performance of the predictive models takes precedence over understanding the relationship between dependent variables and the independent variables”. ML is performed on large amounts of data which reduces likelihood of errors of estimation and measurement. With correlation-based methods, ML aims to reveal patterns instead of causality in “real world data” [23]. For DSTs, profiling data of persons, data of the followed interventions, and data indicating the outcome variable serve as the training set and the validation set. With an algorithmic approach, the DST learns what works for whom. After the algorithm is trained and validated, the DST is implemented in regular service and individual determinants (the profile) can serve as input. Then, the output of the DST is a recommendation for an intervention. The validation set and training set are usually independent, but are often a selected part of the same large dataset [1, 2]. We refer to the introduction of this special series for a more detailed definition and explanation of ML and the main differences with conventional statistical methods [13]. In brief, “many ML algorithms are adopted from the statistics literature, and a number of Bayesian methods have been incorporated. ML usually does not attempt to isolate the effect of any single variable, but is concerned with building an empirical algorithm for purposes of prediction or classification. ML approaches include random forests, recursive partitioning (CART) and decision trees, bagging, boosting, support vector machines, neural networks, deep learning, and others. Many ML approaches do not model the data generating process but rather attempt to learn from the dataset at hand” [13].

In our clinical example, data for development of the ML algorithm were obtained from workers at a variety of companies during care as usual. Data were collected by OHCPs that work for an Occupational Health Service company. With respect for privacy and consent, the ML-DST is developed in co-operation with the Occupational Health Service and research institutions. Data are provided to the ML algorithms as a training-set and a validation-set, predicting suitable interventions to maintain sustained employability as the outcome. After implementation, the ML-DST will continue to develop because it is a learning system where new collected data can constantly be used to train the system and provide more detailed predictions for sustained employability due to the techniques that are used.

## Results

Improving sustainable employment of workers with an efficient, valid support for clinical decision making for use by OHCPs is the reason for developing ML-DSTs with the best available techniques. The ML-DST will induce a change in behaviour and perceptions of stakeholders involved [3, 4, 10]. The ethical issues that emerge are sometimes unique to the new technology, but can also be inherent to an advisory process in general. For example, privacy has always been an issue in using and developing DSTs with conventional methods and statistics. We have described and discussed results with a focus on the changes that occur as a result of the new technology and what this means for autonomy, beneficence, non-maleficence and justice. First, we describe what implications the scenario have for Tom according to the ethical principles. Next, we explain the issues that specifically emerge when using the new technology and how they relate to the ethical principles. For each ethical principle we close with the consequences and possible directions for stakeholders and developers. Table 3 provides an explanation of the key issues identified in the literature and summarizes their relation to the four basic ethical principles.

**Table 3:** Explanation of issues related to the principles in biomedical ethics [8]

Principles in biomedical ethics [8]	Issues identified in the literature related to the principles	How the issues apply to the principles in biomedical ethics when using an ML-DST
Autonomy		
	Consent: “In any research on human beings, each potential subject must be adequately informed of the aims, methods, sources of funding, any possible conflicts of interest, institutional affiliations of the researcher, the anticipated benefits and potential risks of the study and the discomfort it may entail. The subject should be informed of the right to abstain from participation in the study or to withdraw consent to participate at any time without reprisal. After ensuring that the subject has understood the information, the physician should then obtain the subject’s freely-given informed consent, preferably in writing.” [24] Consent for use of personal data is also governed by law [20, 24]	The flexible nature and possible modifications of the ML-DST after implementation, contribute to uncertainty about what future consequences the worker is actually consenting for Autonomy is affected by verifiability of data validity of the ML-DST used to inform the worker. Although increased validity through ongoing use of ML-DSTs may be possible, available evidence is still weak and more research and development is necessary
	Verifiability: Conduct is verifiable when it is possible for others to assess whether it complies with relevant standards (for instance of quality or reliability). [25]	
	Validity: refers to an “unbiased study” that, based on its design, methods, and procedures, will produce (on average) overall results that are close to the truth [26]	
Beneficence		
	Validity: Referred to above	The three elements of evidence based practice are affected by use ML-DSTs, which influences beneficent provision of advice. First, the evidence and data used by the ML-DST constantly changes and a critical stance towards validity is required. Secondly, the OHCP requires new clinical and epistemological expertise to apply recommendations from ML-DSTs. Thirdly, the patient (worker)’s value towards the advice received depends on trust. Therefore, the OHCP should be able to explain and balance results of the ML-DST with their own professional opinion and the specific worker’s circumstances
	Evidence-based medicine: “the conscientious, explicit, and judicious use of current best evidence in making decisions about the care of individual patients. Evidence-based practice is applied by integration of best research evidence with clinical expertise and patient values” [27]	
Non-maleficence		
	Context: Workers are vulnerable in the occupational context because they depend on their job for social participation and salary, but they can be replaceable. [20]	The principle of non-maleficence is challenged when attempting to balance potential group-level benefits with risk of individual harm. The nature of ML techniques allows possible violation of values towards individual privacy. This should be considered regarding the social and financial vulnerability of the worker in his occupational context. The blurred custodianship and ownership of data in combination with privacy issues and the context require cautiousness regarding the possibility of function creep
	Function creep: is the use of techniques for unforeseen and different purposes than the developers aimed for. [7]	
	Privacy: “The right of research subjects to safeguard their integrity must always be respected. Every precaution should be taken to respect the privacy of the subject, the confidentiality of the patient’s information and to minimize the impact of the study on the subject’s physical and mental integrity and on the personality of the subject.” [24]	
	Ownership of the data: The data owner is responsible and accountable for the protection and classification of specific data	
	Custodianship of the data: The data owner can delegate responsibilities related to data ownership to a custodian.	
Justice		
	Profiling: “Gathering information about an individual (or group of individuals) and evaluating their characteristics or behavior patterns in order to place them into a certain category or group, in particular to analyze and/or make predictions about, for example, their ability to perform a task, interests or likely behavior.” [28]	When advice is impartial and objective, people are treated more equally without prejudice, which is beneficial for the worker. However, this only counts when the worker is validly profiled and well represented by the ML-DST. Profiles might lead to discrimination by excluding groups from, for instance, tasks or jobs because of a conflict of interest between an employer’s financial interest and the worker’s health interest
	Discrimination: Prejudiced treatment or consideration of, or making a distinction towards, a person based on the group, class, or category to which the person is perceived to belong, in a way that is disadvantageous for the person. [29]	
	Conflict of interest by OHCPs/employers Refers to impartiality. “Academic practitioners are impartial and objective when they do not let personal interest, preference, affections, prejudice or the interests of the commissioning or funding body affect their judgement and decisions.” [25] Refers to independency: “When presenting insights as correct and relevant, academic practitioners are independent when they only allow themselves to be influenced by others’ judgements to the degree that such judgements are based on scientific or scholarly authority. They do not allow themselves to be influenced on other grounds.” [25]	

### Respect for Autonomy

#### Clinical Scenario

Tom makes his own autonomous decision to improve his health. However, Tom feels resistance to the digitized process and the outcome. He does not want to be pushed in a direction he does not feel comfortable with and he wants to be able to refuse the intervention. A precondition for an autonomous choice is that the choice is well-informed, but both the OHCP and Tom are insecure about the quality of the advice.

#### Issues Discussed: Consent, Verifiability and Validity of Data

To respect Tom’s autonomy, he should have free choice. But a potential adverse effect of the digitized process might be that Tom feels forced into treatment options, which affects his autonomy and personal freedom. Consent is an important principle for protection of this aspect of autonomy, and is partly governed by law [20, 30] and partly by scientific ethical standards [25, 31, 32]. According to the Declaration of Helsinki, health sciences researchers are required to explain the research to participants in a manner they can understand. Informed consent is required for most studies involving human participants, however, may be waived in some situations involving health registries, large databases, or data gathered as part of care as usual in some jurisdictions. In Tom’s case the development of the ML-DST for a WHA used data gathered as part of care as usual and the study was waived by the medical ethical committee. Nevertheless, workers included in the development process signed an open consent [22], meaning they agreed that their data could be used for scientific purposes to improve the health service without specifying the use and they can withdraw whenever they want. In this case, Tom provided consent but it may be unclear if he understood what he consented to. A major difference for ML-based decision support is that the technique is flexible by nature and after initial development the algorithms are regularly updated with new sets of data. The algorithm structure can be adjusted over the course of time to address new emerging themes. Especially when the phase of development fades into implementation of the ML-DST as part of usual care, modifications might no longer be registered. Additionally, the analytic process and the inferred classification model underlying recommendations often become non-transparent and non-verifiable.

Another important part of an autonomous choice is that the worker is well informed. Tom should be able to rely on the professional’s advice as valid and accurate. There are two aspects to address. First, the objective quality of the ML-DST output and second the advice given by the professional. This last aspect will be covered in the paragraph “beneficence”, the objective quality of the ML-DST output will be explored in the following paragraphs.

Validity of research indicates the extent to which results are representative, accurate, and reliable for the group and for the purpose studied. Verifiability of data is a prerequisite for reliability. ML has the potential to provide better information for decision making than traditional specialists, reducing the likelihood of human error, and increasing external validity [21, 23]. This would be a great benefit for all potential users of ML-DSTs. Reasons provided for better classification and prediction are the large amount of data the outcome is based on that reduces likelihood of errors of estimation and measurement, the advanced techniques used, the shift from causality based research to correlation based research revealing patterns that people often are not aware of, and the use of “real world” data instead of controlled data from clinical study designs [23]. Each of these theoretically contribute to providing a better picture of the real situation, which is one of the main reasons to use ML techniques. The validity of the advice and recommendations made would improve, which would contribute to Tom’s health and wellbeing.

Unfortunately, the positive idea that real world data informs development of ML-DSTs to provide improved validity should be tempered by the knowledge that the output of ML algorithms depend on the choices made for input-data and algorithms used [9, 33]. Achieving valid and replicable classification models is currently challenging [9], and algorithms may not be as valid as hoped for. Often models are theory-based or made and constrained by humans who do not necessarily have (bio-)medical knowledge [21, 34]. Beyond this, the technical choices in deep learning methods have a black box character and are not transparent. Often the decision paths are too complex to be understood by humans [34]. Although deep learning methods often provide better accuracy [13], findings could emerge that are not directly related to the study and clinical significance of findings is not always obvious [22]. A consequence of this lack of verifiability or transparency is that results may be invalid without users even being aware.

#### Consequences and Directions for Stakeholders and Development

Participants have the right that any consent they provide represents an informed choice, and therefore, they should be informed about future use and consequences of the processing of their data by researchers and users (e.g., OHCPs) [28]. The application of ML-techniques simplifies adjustments to algorithms and can allow the original purpose of data gathering to be changed. When assessing a research application, Health Research Ethics Committees should be aware of and address possible changes of the ML-DST characteristics (e.g., input parameters, algorithm) after the developmental phase. To our knowledge there is increasing awareness and guidelines are being developed [28], but a standardized approach to ethical approval and oversight has not yet been specified for all Ethics Committees yet.

While using ML-DSTs, respect for autonomy requires disclosure of information that is valid, reliable and transparent. ML techniques are theoretically able to provide better predictions than humans or traditional statistical methods, and are therefore essentially a positive contribution to a well-informed decision. However, this is not always the case, as demonstrated by a validation study [9] where results of replication were unfavorable. A change in context (e.g., insurance policy or legislation) might dramatically influence the validity of results. Although this could be corrected over time when feedback loops suggest that previous recommendations are not successful anymore, it might also be necessary to include new parameters as input. As mentioned in the method section, the validation dataset used for development is often part of a large dataset. Validation outside the primary development dataset would improve knowledge about external validity. This indicates the importance for experts from the various domains to collaborate, whereby the medical experts can ensure that the input is correct and that the Information Technology (IT) specialist make the connections using the right methods. Additional clinical research is then needed to evaluate the output and algorithm changes.

### Beneficence

#### Clinical Scenario

Tom would like the provided advice to fit his needs, which it does not. The OHCP disagrees with parts of the ML-DST output. However, the ML-DST was developed using state of the art techniques to provide the best available, valid options for possible interventions for Tom. What benefits Tom most?

#### Issues Discussed: Validity, Evidence Based Practice

To address the principle of beneficence, the OHCP aims to support workers in their sustainable ability with “the conscientious, explicit, and judicious use of current best evidence in making decisions about the care of individual patients” [27]. Interaction with the new ML-DST technique brings insecurity to the decision making process. Although the technical approaches might improve, people may experience them differently, which influences the value of beneficence. Evidence-based medicine is applied through integration of best *research evidence* with *clinical expertise* and *patient values* [27]. With the introduction of the new ML technology, the principle of evidence based medicine remains, but the three elements change. First, the research evidence changes. Compared to traditional WHAs, application of an automated WHA based on ML has great potential to advance prevention efforts and foster healthy habits [35]. But the ML-DST advice strongly depends on the validity of the algorithm and recommended results. As outlined in the paragraph about respect for autonomy, there are currently reasons for a critical stance towards the validity of ML-DST algorithms, needing interdisciplinary collaboration and a critical approach to development of the DST and to maintaining protecting integrity of the database. Secondly, the clinical expertise changes. Professional competence is built on knowledge, experience with former clients, clinical reasoning, communication and decision making [36]. The OHCP is constantly learning and adjusting, comparing options using an autonomous internal neural network. This same learning process is implemented in the development ML-DST, so in theory ML-DSTs may be trained to learn, develop, and make decisions like a competent and successful OHCP. However, a main difference between the “internal network of an OHCP” and the ML-DST network is that the advice of an ML-DST now informs and influences OHCP decisions, with unknown consequences. The third and last element that changes are patient values. Trust is a precondition for beneficence. The way the worker values the advice received relates to the trustworthiness of the professional [34, 37]. How can the worker trust the ML-DST or the OHCP when the results cannot be explained? Generally, there is a lack of understanding about ML techniques among OHCPs and their clients (workers), which can be an obstacle to trust in decisions informed by ML algorithms [34]. It is important that results are interpreted in the context of all available evidence, which also implicates knowledge about the ML algorithm development [22].

#### Consequences and Directions for Stakeholders and Development

A better knowledge and understanding of the OHCP related to ML-DSTs could improve trust in the ML-DST with beneficence as a consequence. The OCHP relying on the results of the ML-DST should be familiar with the choices made in advance and their consequences, then take them into account in the interpretation to provide a judicious decision [34]. This requires epistemological knowledge about the ML-DST as well as a critical and reflective appraisal of the traditionally acquired clinical knowledge of the professional. It is also important the OHCP is able to explain the decisions and the process to the worker. The required new knowledge and different skills give new meaning to the professional role [22, 38, 39].

### Non-maleficence

#### Clinical Scenario

Tom is afraid that his data will be shared with third parties against his will. He is worried that the OHCP will share results with his employer who might judge him as unfit for his job. He is also afraid the data and results will be shared with insurance companies and have a negative impact on his health insurance premium.

#### Issues Discussed: Context, Function Creep, Privacy, Custodianship and Ownership

The occupational context for developing and using an ML-DST brings great opportunities as well as considerations and complications from an ethical perspective. [20] Many stakeholders with often conflicting priorities are involved: e.g., the employer, the employees, regulations, the OHCP, software-developers, researchers and insurance companies. Although all intentions might be good, they often have different interests and perspectives. An important consideration for this setting is the vulnerability of the worker and the potential harm an ML-DST may cause [20, 28].

The use of techniques for unforeseen and different purposes than the developers aimed for is called function creep [5]. When personal data are involved, privacy becomes an issue and misuse might bring harm. In case of the ML-DST for a WHA, the tool might be used for exclusion of workers, based on pre-assumptions about the health risks that will be revealed. This is shown in our scenario where Tom is afraid to lose his job. Local law, regulations, codes and rules of conduct should bring clarity and help ensure patient privacy is protected. However, due to technological and societal developments, these privacy rights have become ambiguous. Examples of unforeseen use of data are already available. Mendelson et al. [40] described an Australian situation where health records were co-linked lawfully with new technology, but the result was that third parties could access individual’s health information without the patient’s knowledge or consent [40]. Even in a closed environment without sharing of databases, the custodianship and ownership of data might become blurred. In our scenario, people who change jobs or stop working remain in the database and it is difficult to delete their information from the database if requested [41]. This becomes even more challenging when data originate from different companies. These examples of function creep with health data show how important it is that we are aware and that we have to reflect on how we value privacy, custodianship and ownership of data.

The risk of individual harm and the right to privacy, custodianship and ownership of personal data should be balanced with the potential benefits for the group when data are shared. It might be argued that the improvement of health on a group-level should outweigh how we value privacy in relation to autonomy for an individual. This is in line with the societal movement of the choice for openness and sharing of data [21, 22]. A profound development has been the public’s willingness to share personal information in a variety of public venues (e.g., social media, wearable devices and health apps). Furthermore, many societies (e.g. United States of America, China, European Union) have embraced ‘big data’ research for health science in parallel to the increasing transparency and public accountability that governments are moving towards [22].

#### Consequences and Directions for Stakeholders and Development

A realistic concern is group-level harm or individual harm in case of function creep, because the workers are vulnerable in relation to their employer. In our opinion, it is a responsibility of the developers to take this into account when developing ML-DSTs. It is also a responsibility of users to respect integrity and balance the risk of harm for an individual with the potential beneficence for the group. The privacy, custodianship and ownership issues do not uniquely apply to our scenario, because the sharing of personal information is more integrated into our daily lives than we are aware of, through the application of technology. It is critically important to sufficiently consider the consequences for our freedom, and carefully consider the consequences to non-maleficence, autonomy and justice.

### Justice

#### Clinical Scenario

Tom is afraid that the results of his WHA and the matching advice will compromise his position at the company and maybe even his options for health insurance. Besides, the profile that was created was not congruent with his own image of his essential health issues and he didn’t feel recognized as a person. Tom is afraid that workers with the same constitution (e.g. gender, age, weight, vitality) who did not attend the assessment will be favoured over him.

#### Issues Discussed: Profiling, Discrimination and Conflict of Interest on the Part of OHCPs/Companies

The models created and used by ML-DSTs lead to profiles of workers for whom an intervention could be successful. This is inherent to the main goal of the ML-DST: to identify patterns and build classifications from available person characteristics, health services information, as well as work-related and health outcomes. ML techniques theoretically provide objective results, free of prejudice about the person, to render equitable recommendations. However, beyond the validity of the classifications, workers may have unique characteristics that are difficult to measure and include in databases, causing bias as a result of omitted variables. Questions may arise as to whether the ML-DST process does justice to the individuals being profiled as members of a category that is separate from the individual’s unique identity [42]. The phenomenon is not different from issues that arise from applying classical clinical practice guidelines, but the perception might be different when the ML-DST is presented as “personalized” [42]. On the other side of the spectrum, when profiling becomes very detailed and many variables are used for identification, anonymity might be at risk. The right to privacy as the right not to share information was already addressed. Here the consequences of being recognized at the expense of equal treatment is explored. Several studies have highlighted that the techniques for ML have reached a level of sophistication that makes it impossible to promise perfect anonymity, even when data are depersonalized [22, 23, 42]. These risks apply particularly when data used in analyses are from open sources [22], such as social media, device location, sports apps. For an occupational context with identifiable parameters such as job, task or years employed, this might also be a major risk. In this case, Tom is afraid for his position at the company and being discriminated against in favor of colleagues who did not participate in the assessment.

Another problem with group profiling is that the ML-DST for sustained employability could be used for discrimination [28]. For example, by excluding people from work on the group-level [22] when recommended interventions are not cost-effective. Profiling and discrimination might put people into different positions leading to social and potentially even economic inequality, which detriments judicious treatment. Even the possibility that this could happen might create a feeling of insecurity among workers, as shown in the example of Tom. This would affect how the worker behaves and therefore also affect his autonomy, the first value that was discussed.

Although the risks of denying an individual’s identity, the possibility of discrimination, and detailed profiling are also possibilities with care-as-usual situations, the theoretical performance capabilities of ML techniques increase the chance it might actually happen. Mittelstadt and Floridi [42] and Lipworth et al. [22] have identified this increased risk of misuse as a reason for users to value advice provided by ML-DSTs differently [22, 42] than traditionally generated advice from OHCPs. The profiling might influence the interpretation and decisions of the OHCP and negatively affect the relationship between the OHCP and the worker.

#### Consequences and Directions for Stakeholders and Development

The potential benefits for workers from using ML-DSTs in occupational health are evident and the objectivity of the tools may actually increase the likelihood of equal treatment without prejudice. However, this reasoning only counts if the profiles actually represent the person correctly. Researchers and users of ML-DSTs should take joint responsibility to ensure that profiles will not lead to discrimination of individuals, however this may lead to lower predictive values of the ML-DST. The issues related to profiling and potential for discrimination should also be formally evaluated when Health Research Ethical Committees assess research applications [23].

## Discussion and Implications

We deliberated the ethical implications of using an ML-DST in occupational health care by reflecting on the principles of respect for autonomy, beneficence, non-maleficence and justice [8]. Questions arose that affect both client and professional values, leading to important ethical dilemmas for this area of practice. One major characteristic of an ethical dilemma is when two opposing values or principles occur at the same time: both are ideals we pursue but cannot ultimately reach [8, 43]. For example: what if the outcome recommended by the ML-DST is objectively beneficial for the worker, but the worker chooses not to follow the advice? Not all of the dilemmas are a direct result of using the technology, but we explored how the technology might influence values and dilemmas. In line with this, issues we explored often relate to more than one ethical principle. For instance, discrimination affects a fair opportunity for people, affecting the principle of justice. However, it can also do harm when pre-assumptions about health result in exclusion from job tasks, affecting the principle of non-maleficence. This paper does not address how to deal with dilemmas but gives insight into what might happen and how to anticipate them.

Technical, methodological and epistemological aspects of the ML technique and the decisions made for development appear to have ethical implications. It is out of scope of this manuscript to give detailed directions for ML techniques and methodology for development and validation of DSTs. Instead we recommend considering the impact and potential adverse effects of the ML-DST at different phases of the innovation process, incorporating input from multiple disciplines for a socially responsible design.

Although our aim was to explore emerging universal ethical issues of using an ML-DST in the occupational context, subjectivity could not be avoided because values are not consistent across all cultures. As Ebbesen et al. [43] point out, there are reasons to assume that the values as formulated by Beauchamp and Childress [8] do not reflect universal morality, but the characteristics of North American society. We chose this classification because it is most commonly used in the biomedical context and, according to Beauchamp and Childress, it reflects common morality beyond the medical world including the occupational context. Another culture-specific classification might bring other ethical issues and dilemmas to light.

Summarizing the consequences and directions for stakeholders that were discussed in the results, three recommendations for future ML-DST development arise. Although this cannot fully prevent unforeseen ethical issues, applying this advice should contribute to minimizing undesirable adverse effects.

- The first recommendation is the importance of ‘educating’ the ML-DST well by providing it with the best available data, training it with the best available algorithms, validating the ML-DST, and discussing its ethical impact. Including input from all relevant stakeholders during development and implementation (e.g., health care professionals, IT specialists, employees, employers, researchers) will assist in considering the possible impact and side effects. This will enable socially responsible design and a valid and ethically-accepted ML-DST, while minimizing risk of function creep.
- Secondly, ML-DST research applications should be formally assessed by Health Research Ethical Committees for the risk of potential profiling, function creep, discrimination as well as issues around privacy, custodianship and ownership of the data and the new tools developed. To our knowledge this is a new domain for Research Ethics Committees, therefore awareness and new approval and oversight procedures are likely required. Considerations should be specific to individual occupational settings where the studies are being conducted.
- Thirdly, OHCPs should be educated about the epistemology of the ML-DST and their strengths and weaknesses to enable conscientious, explicit, and judicious use of current best evidence in making decisions about the care of individual patients while being informed by these new potentially beneficial techniques [27].

These recommendations arose from our example of a workers’ health assessment, but likely also apply to other occupational health-related activities were ML-DSTs are introduced and the evidence based practice will be informed by these techniques. To continue the necessary, trustful relationships with clients it is an essential prerequisites that users (OHCPs) are educated and ethical values are balanced and protected by all stakeholders.

## References

[1] Gross DP, Zhang J, Steenstra I, Barnsley S, Haws C, Amell T (2013). Development of a computer-based clinical decision support tool for selecting appropriate rehabilitation interventions for injured workers. J Occup Rehabil.

[2] Fong J, Ocampo R, Gross DP, Tavakoli M (2020). Intelligent robotics incorporating machine learning algorithms for improving functional capacity evaluation and occupational rehabilitation. J Occup Rehabil.

[3] Verbeek PPCC (2005). What things do: philosophical reflections on technology, agency and design.

[4] Swierstra T (2015). Identifying the normative challenges posed by technology’s ‘soft’ impacts. Etikk i praksis Nordic J of Appl Eth..

[5] Dorrestijn S, van der Voort M, Verbeek P-P (2014). Future user-product arrangements: combining product impact and scenarios in design for multi age success. Technol Forecast Soc Change.

[6] Liebert W, Schmidt JC (2010). Towards a prospective technology assessment: challenges and requirements for technology assessment in the age of technoscience. Poiesis Praxis.

[7] Long TB, Blok V, Dorrestijn S, Macnaghten P (2019). The design and testing of a tool for developing responsible innovation in start-up enterprises. J Responsib Innov.

[8] Beauchamp T, Childress J (2009). Principles of biomedical ethics.

[9] Gross DP, Steenstra IA, Shaw W, Yousefi P, Bellinger C, Zaiane O (2019). Validity of the Work Assessment Triage Tool for selecting rehabilitation interventions of workers’ compensation claimants with musculoskeletal conditions. J Occup Rehabil.

[10] Dorrestijn S (2012). Our hybrid selves: figures of technical mediation (ethical substance). The design of our lives—technical mediation and subjectivation after Foucault.

[11] Dorrestijn S, Niedderer K, Clune S, Ludden G (2017). The product impact tool: the case of the Dutch public transport chip card. Design for behaviour change: theories and practices of designing for change.

[12] Tromp N, Hekkert P, Verbeek P-P (2011). Design for socially responsible behavior: a classification of influence based on intended user experience. Des. Issues.

[13] Gross DP, Steenstra IA, Harrell Jr FE, Bellinger C, Zaiane O. Special series on machine learning for work disability prevention: definitions and key issues. J Occup Rehabil. (in press) 10.1007/s10926-020-09910-1 32623556

[14] Page K (2012). The four principles: can they be measured and do they predict ethical decision making?. BMC Med Ethics.

[15] Rawls J (1971). A theory of justice.

[16] WHO Regional Office for Europe (2002). Good practice in occupational health services: a contribution to workplace health.

[17] van Holland BJ, Soer R, de Boer MR, Reneman MF, Brouwer S (2015). Workers’ health surveillance in the meat processing industry: work and health indicators associated with work ability. J Occup Rehabil.

[18] Weel ANH, Duijn JCM, van Vliet C (2007). Preventive Workers’ Health Surveillance: Dutch language translation. TBVG.

[19] Six Dijkstra M, Soer R, Bieleman A, McCraty R, Oosterveld F, Gross D (2019). Exploring a 1-minute paced deep-breathing measurement of heart rate variability as part of a Workers’ Health Assessment. Appl Psychophysiol Biofeedback.

[20] International Labour Organization (1998). Technical and ethical guidelines for Workers’ Health Surveillance. Occupational Safety and Health Series (72).

[21] Estape ES, Mays MH, Sterke EA (2016). Translation in data mining to advance personalized medicine for health equity. Intell Inf Manag.

[22] Lipworth W, Mason PH, Kerridge I, Ioannidis JPA (2017). Ethics and epistemology in big data research. J Bioeth Inq.

[23] Ienca M, Ferretti A, Hurst S, Puhan M, Lovis C, Vayena E (2018). Considerations for ethics review of big data health research: a scoping review. PLoS ONE.

[24] World Medical Association. WMA Declaration of Helsinki—ethical priciples for medical research involving human subjects. 2013. https://www.wma.net/policies-post/wma-declaration-of-helsinki-ethical-principles-for-medical-research-involving-human-subjects/. Accessed 16 Oct 2019.

[25] ALLEA. The European code of conduct for research integrity—revised edition. Berlin: ALLEA: All European Academies; 2017.

[26] Szklo M, Nieto F (2007). Epidemiology, beyond the basics.

[27] Sackett DL, Rosenberg WM, Gray JA, Haynes RB, Richardson WS (1996). Evidence based medicine: what it is and what it isn’t. BMJ.

[28] Guidelines on Automated individual decision-making and Profiling for the purposes of Regulation 2016/679. WP251rev01. Brussels: Directorate C of the European Commission Directorate General Justice; 2018 (revision). https://ec.europa.eu/newsroom/article29/item-detail.cfm?item_id=612053. Accessed 16 Oct 2019.

[29] Horta O (2010). Discrimination in terms of moral exclusion. Theoria: Swed J Philos.

[30] Regulation (EU) 2016/679 of the European Parliament and of the Council of 27 April 2016 on the protection of natural persons with regard to the processing of personal data and on the free movement of such data, and repealing Directive 95/46/EC (General Data Protection Regulation). The European Parliament and the Council of the European Union. 2016. https://eur-lex.europa.eu/legal-content/EN/TXT/PDF/?uri=CELEX:32016R0679. Accessed 16 Oct 2019.

[31] Groningen UMC (2018). Research code.

[32] National Academies of Sciences, Engineering and Medicine (2017). Fostering integrity in research.

[33] Qin Z, Armijo-Olivo S, Woodhouse LJ, Gross DP (2016). An investigation of the validity of the Work Assessment Triage Tool clinical decision support tool for selecting optimal rehabilitation interventions for workers with musculoskeletal injuries. Clin Rehabil.

[34] Watson DS, Krutzinna J, Bruce IN, Griffith CEM, McInnes IB, Barnes MR (2019). Clinical applications of machine learning algorithms: beyond the black box. BMJ.

[35] Costa FF (2014). Big data in biomedicine. Drug Discov Today.

[36] van Merriënboer JJG, Clark RE, de Croock MBM (2002). Blueprints fo complex learning: the 4C/ID-model. Educ Technol Res Dev.

[37] Lipton Z. The doctor just won’t accept that! Interpretable ML symposium, 31st conference on neural information processing systems (NIPS 2017); 2017; Long Beach, CA, USA.

[38] Yost J, Dobbins M, Traynor R, DeCorby K, Workentine S, Greco L (2014). Tools to support evidence-informed public health decision making. BMC Public Health.

[39] Pope C, Halford S, Turnbull J, Prichard J, Calestani M, May C (2013). Using computer decision support systems in NHS emergency and urgent care: ethnographic study using normalisation process theory. BMC Health Serv Res.

[40] Mendelson DR, Rees A, Wolf G. Medical confidentiality and patient privacy. Chapter 9. In: White B, McDonald F, Willmott L, editors. Health Law in Australia, 3d ed. Thomson Reuters; 2018; 9.10-9.280. https://ssrn.com/abstract=3173601. Accessed 16 Oct 2019.

[41] Zarate OA, Brody JG, Brown P, Ramirez-Andreotta MD, Perovich L, Matz J (2016). Balancing benefits and risks of immortal data: participants’ views of open consent in the personal genome project. Hastings Cent Rep.

[42] Mittelstadt BD, Floridi L (2016). The ethics of big data: current and foreseeable issues in biomedical contexts. Sci Eng Ethics.

[43] Ebbesen M, Andersen S, Pedersen BD (2012). Further development of Beauchapm and Childress’ theory based on empirical ethics. J Clin Res Bioeth.

